# Unsupervised Clustering-Assisted Method for Consensual Quantitative Analysis of Methanol–Gasoline Blends by Raman Spectroscopy

**DOI:** 10.3390/molecules29071427

**Published:** 2024-03-22

**Authors:** Biao Lu, Shilong Wu, Deliang Liu, Wenping Wu, Wei Zhou, Lei-ming Yuan

**Affiliations:** 1 School of Information and Engineering, Suzhou University, Suzhou 234000, China; 2 Suzhou Vocational and Technical College, Suzhou 234000, China; 3 College of Electrical and Electronic Engineering, Wenzhou University, Wenzhou 325035, China

**Keywords:** Raman spectroscopy, methanol–gasoline blends, partial least squares discriminant analysis (PLS-DA), self-organizing mapping (SOM), consensus model

## Abstract

Methanol–gasoline blends have emerged as a promising and environmentally friendly bio-fuel option, garnering widespread attention and promotion globally. The methanol content within these blends significantly influences their quality and combustion performance. This study explores the qualitative and qualitative analysis of methanol–gasoline blends using Raman spectroscopy coupled with machine learning methods. Experimentally, methanol–gasoline blends with varying methanol concentrations were artificially configured, commencing with initial market samples. For qualitative analysis, the partial least squares discriminant analysis (PLS-DA) model was employed to classify the categories of blends, demonstrating high prediction performance with an accuracy of nearly 100% classification. For the quantitative analysis, a consensus model was proposed to accurately predict the methanol content. It integrates member models developed on clustered variables, using the unsupervised clustering method of the self-organizing mapping neural network (SOM) to accomplish the regression prediction. The performance of this consensus model was systemically compared to that of the PLS model and uninformative variable elimination (UVE)–PLS model. Results revealed that the unsupervised consensus model outperformed other models in predicting the methanol content across various types of methanol gasoline blends. The correlation coefficients for prediction sets consistently exceeded 0.98. Consequently, Raman spectroscopy emerges as a suitable choice for both qualitative and quantitative analysis of methanol–gasoline blend quality. This study anticipates an increasing role for Raman spectroscopy in analysis of fuel composition.

## 1. Introduction

With the development of automobile manufacturing, transportation, and petrochemical industries, the demand for fossil fuels such as oil and coal has increased significantly. This excessive utilization of fossil energy not only contributes to global warming but also poses a significant threat to our planet’s sustainability. Given that fossil energy resources are finite and non-renewable, humanity faces the looming dual challenges of an energy crisis and an environmental crisis in the near future. In response to these pressing issues, nations worldwide are actively exploring environmentally sustainable and economically viable alternative energy sources [1], including wind [2], tidal [3], bio-fuels [4,5], etc.

Among bio-fuels, ethanol and methanol stand as the two preferred alcohol-based bio-fuels. Alcoholic gasoline blends are formulated by mixing gasoline with specialized additives and either ethanol or methanol. This process not only alleviates the shortage of gasoline resources, but also effectively reduces the content of toxic substances in automobile exhaust gas [6,7]. Compared to ethanol–gasoline blends, methanol–gasoline blends have the advantage of lower prices and a higher octane number of 112 than the motor octane number of 106 [7,8,9,10]. Therefore, the blend of methanol and gasoline can enhance the octane number of gasoline, boost its oxygen content, facilitate more complete combustion, and optimize fuel utilization. Given their excellent quality and suitable economic viability, methanol–gasoline blends have great development value in the field of new energy.

However, the methanol content in methanol–gasoline blends is strictly regulated. Typically, the allowed methanol proportion in these blends ranges from 0% to 80%, with narrower limits for certain specialized applications [11,12,13,14]. Deviations from this range, either excessive or insufficient methanol content, can lead to engine damage and inadequate heating [14,15]. Additionally, there is growing demand for on-site analysis of methanol content within methanol–gasoline blends [11,16]. Therefore, it is crucial to develop a rapid sensing method for accurately detecting methanol content. This approach not only ensures effective quality control of methanol–gasoline blends, but also prevents unethical traders from illicitly profiting by selling methanol gasoline.

Numerous methods have been reported for detecting the methanol content in methanol–gasoline blends, including quality assurance/control approaches [14], physical–chemical methods [5], chromatographic methods [9] and spectroscopic methods [17,18,19,20]. Chromatographic analytical techniques, such as high-performance liquid chromatography (HPLC) and gas chromatography mass spectrometry (GC-MS) serve as precise and standardized measurements of methanol content [21]. Nevertheless, they come with certain drawbacks, including labor-intensive procedures, time consumption, and high costs. Furthermore, compared to infrared or Raman spectroscopy [9,16,22], their most significant limitation is the inability to perform timely on-site analysis. In addition, these methods require complex sample pretreatment and the use of toxic and hazardous reagents, posing health risks to operators. Fortunately, the advancement and application of infrared/Raman spectroscopy have effectively overcome many of the limitations associated with gas chromatography [19,22,23].

Raman spectroscopy, as an efficient and fast spectral analytical technique, has been widely utilized in fields such as food, medicine, chemistry, physics, biology, and others [23,24,25,26]. Its non-destructive nature and potential portability, make it suitable for read-time detection in various situations, providing valuable information on the composition of fuel components [23,24]. Raman spectroscopy can directly represent the molecular vibration information of various functional groups, such as olefins and aromatic hydrocarbons in gasoline fuel [23]. Furthermore, compared to near-infrared or mid-infrared spectroscopy, Raman spectroscopy has the advantage of being unaffected by moisture. Since some absorption characteristics of molecules in near-infrared spectroscopy are overlapped by other alcohols, spectral signals are obscured in methanol gasoline [16,22]. Raman spectroscopy can avoid these issues, making it particularly suitable for methanol gasoline qualitative or quantitative analysis, respectively [16,22,27].

**Figure 1:**
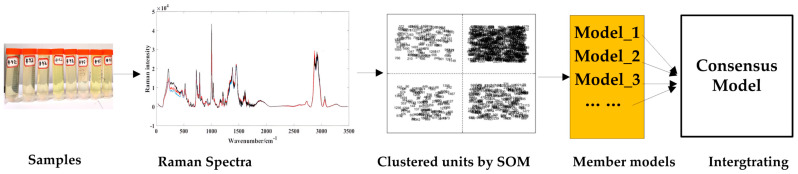
Workflow of the modeling method.

In this study, qualitative and quantitative analysis are combined to analyze the quality of methanol gasoline blends. The three sections are organized as follows: (1) qualitative classification studies of gasoline types (92#, 95#, 98#) are carried out using partial least squares–discriminant analysis (PLS-DA); (2) the similar characteristic variables in Raman spectra of methanol–gasoline blends are clustered by the unsupervised variable clustering method of the self-organizing mapping neural network (SOM); (3) based on the clustered units obtained from the SOM analysis, separate quantitative models are established. And then, a consensus model is integrated from these individual quantitative models to analyze the methanol content in methanol–gasoline blends, and for comparison, UVE [28,29] is also used to establish the PLS model. Among these processes, the last two parts for quantitative analysis are carried out as Figure 1 shows.

## 2. Theories and Algorithms

### 2.1. Classification Model

Partial least squares discriminant analysis (PLS-DA) serves as a variant classifier derived from the partial least squares (PLS) regression algorithm. Widely adopted as a classification tool in various applications, PLS-DA is particularly effective in scenarios involving two classes [30]. This method builds on the foundation of the PLS regression algorithm, leveraging its capabilities for linear regression in the context of spectral data and categorical variables. The primary goal of PLS-DA is to extract multiple dependent variables from the original data in a way that maximizes the covariance between these variables and the response variable (in this case, the class labels). This process involves identifying the optimal latent variables (LVs), which are combinations of the original predictor variables that best explain the variation in the response variable. These LVs are then utilized to construct a predictive classifier that facilitates the discrimination between distinct classes within the data set. Notably, sample labels are assigned based on a classification threshold determined through statistical methods, such as Bayesian theory [30,31]. The strength of PLS-DA lies in its ability to handle collinearity and noise in the predictor variables, making it suitable for spectral data analysis where such issues are often encountered. Additionally, by focusing on the latent variables that best explain the variation in the response variable, PLS-DA can often achieve better classification performance than traditional discriminant analysis methods.

Taking the example of classifying two classes labeled as 0 and 1, the classification criteria are explicitly defined as: (1) If Ypre (categorical variable value of the verification set) exceeds 0.5 and the deviation is less than 0.5, the sample is determined to belong to a specific class. (2) Conversely, if Ypre is less than 0.5 and the deviation is less than 0.5, the sample is deemed not to belong to the other class. (3) An instability in the discriminant model is indicated when the deviation surpasses 0.5. The capacity of samples in each class should be appropriate, and imbalanced class sizes can lead to biases in the model. This approach ensures a robust and reliable classification process that allows for effective sample categorization based on the classification thresholds determined [31].

### 2.2. Variables Selected by UVE

Instrumental artificial operations and environmental fluctuations have the potential to introduce unpredictable noise into spectra data. In the formulation of spectral analytical models, common strategies involve the utilization of spectral pretreatments and variable selection techniques. These approaches aim to mitigate the impact of noise factors and concurrently reduce the number of variables incorporated into the models. To simultaneously optimize both calculation speed and model performance, this work introduces the uninformative variable elimination (UVE), a classical approach that discerns valuable information based on the stability of variable regression coefficients [29,32]. Within the UVE process, random noise matrices are systematically introduced to the spectra, and a series of regression coefficient matrices are constructed through the establishment of partial least squares (PLS) models, whose latent variables were generally determined by the minimized RMSECV (root mean squared errors of cross-validation). Employing a predefined noise threshold, variables displaying stability values of the regression coefficient below this threshold are excluded, whereas those that exceed it are retained [33]. When Raman spectra profiles of substances such as gasoline, methanol, or their blends are scrutinized, it becomes apparent that these high-dimensional spectra, frequently exceeding 1000 dimensions, inherently contain a certain level of noise. This inherent noise can significantly hamper the predictive capabilities of the developed models. During the UVE optimization process, noise matrices are typically generated with half the dimensions of the input spectral variables. There are no strict regulations regarding the dimensions of the noise matrices, and they are usually optimized through exploration. In this work, the column dimension of the random matrix set to 700.

### 2.3. Variables Clustered by SOM

In this research, the clustering method was utilized not for grouping similar samples, but for aggregating analogous features or variables of samples via specific mapping rules or projection techniques. Unlike methods such as K-means, hierarchical clustering, and PCA loadings, the self-organizing map (SOM) is a proficient method for illustrating the connections between individual points in a spectrum. The SOM network comprises a competitive, iterative and interactive layer, capable of categorizing vectors into multiple clusters equal in number to the neuron nodes. The SOM not only projects multidimensional variables into a lower-dimensional space while preserving topological structure, but it also excels at performing non-linear mappings between input variables [32,34].

In this study, Kohonen SOM was employed to cluster informative wavelengths in laser-induced breakdown spectra, following the winner-takes-all principle, primarily adjusting weights during the training phase. Before initiating the network, the input matrix was transposed to group similar variables rather than sample categories. Consequently, spectral points that share similar characteristics are grouped into the same cluster unit. Although several controlled parameters in the SOM network were systematically examined, some (for example, neighborhood distance, rate tuning, ordering phase learning rate, and steps) had a negligible impact on the clustered outcomes [34]. Therefore, default settings were maintained, with specific optimization for the topology function gridtop and the distance function mandist. Dimensions (specifically, size *q*) of the SOM map play a pivotal role in defining both the number of clustering units and the resultant clustering. Given that the SOM network operates as an unsupervised clustering technique, the clustering outcomes require further validation through subsequent supervised classification or regression methodologies.

### 2.4. Steps of Consensual Quantitative Modeling

The fundamental concept underlying the consensus fusion model is to amalgamate the prediction capabilities of multiple member models to establish the final prediction performance, characterized by minimal prediction errors and heightened accuracy. By integrating the predictive strengths of all member models, as opposed to relying on any single individual model, the consensus fusion model mitigates dependence on specific member models [35,36]. This not only diminishes the risk of overfitting, but also enhances overall robustness. Consequently, the consensus fusion model presents a significant advantage, particularly when faced with datasets characterized by a limited number of samples but high dimensions.

The unsupervised consensus model cleverly combines the strengths of both the SOM algorithm and the foundational consensus fusion model. In this approach, the *N*^2^ clusters generated by the SOM algorithm serve as basic member models. Ultimately, the prediction performance of these *N*^2^ member models is weighted to formulate the predictive performance of the unsupervised consensus model. This integration allows for a comprehensive and synergistic utilization of the SOM algorithm’s clustering capabilities and the consensus fusion model’s prowess in synthesizing diverse predictions. The basic steps are the following.

(1) Take the sample matrix *X* as the input of Kohonen SOM network, the variables are clustered as Equation (1), which shows a union *X* of each *k*th cluster subset *X_i_*. If *q* = 2, the Kohonen SOM generates *q*^2^ clusters.

(1) $$X=\{x_{1},x_{2},x_{3},\cdots ,x_{k}\},k=1,2,\cdots ,q^{2}$$

(2) Construct PLS models based on the set of clustered variables $x_{k},\;k=1,2,\cdots ,q^{2}$, respectively, and obtain the prediction result $y_{k}=f_{k}(x_{k}),\;k=1,2,\cdots ,q^{2}$.

(3) Develop a consensus model as Equation (2)

(2) $$F(x)=c_{1}\cdot f_{1}(x_{1})+c_{1}\cdot f_{2}(x_{2})+\ldots +c_{n}\cdot f_{n}\left(x_{n}\right)=\sum_{i=1}^{n}c_{i}\cdot {\hat{y}}_{i}$$

where $c_{i}$ is weightings and can be determined using non-negative least squares constraints, and it can also be used to solve generalization and relevance issue.

(4) Combine the predicted results of the constructed member models with the weightings as the prediction result of the unsupervised consensus model, and the constraints of $c_{i}$ are shown in Equation (3). More theoretical calculations on the consensus fusion model can be found in our previous work [35].

(3) $$\left\{\begin{array}{l} ARG\min\left\{\sum_{i=1}^{4}{{(c}_{i}(y_{n}-y_{i}(x_{i})))}^{2}\right\}\\ 0\leq \left|c_{i}\right|\leq 1\\ \sum_{i=1}^{4}c_{i}=1 \end{array}\right.$$

## 3. Results and Discussion

### 3.1. Analysis of the Raman Spectral Feature

**Figure 2:**
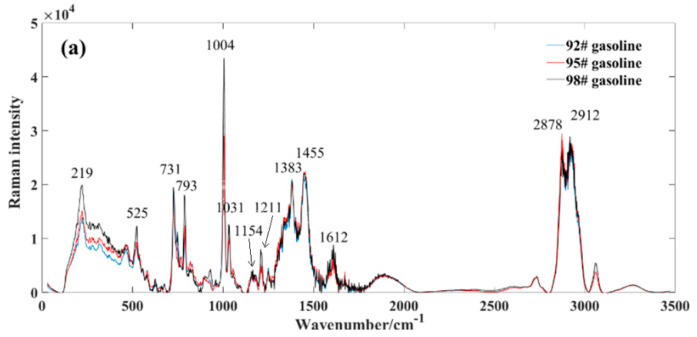

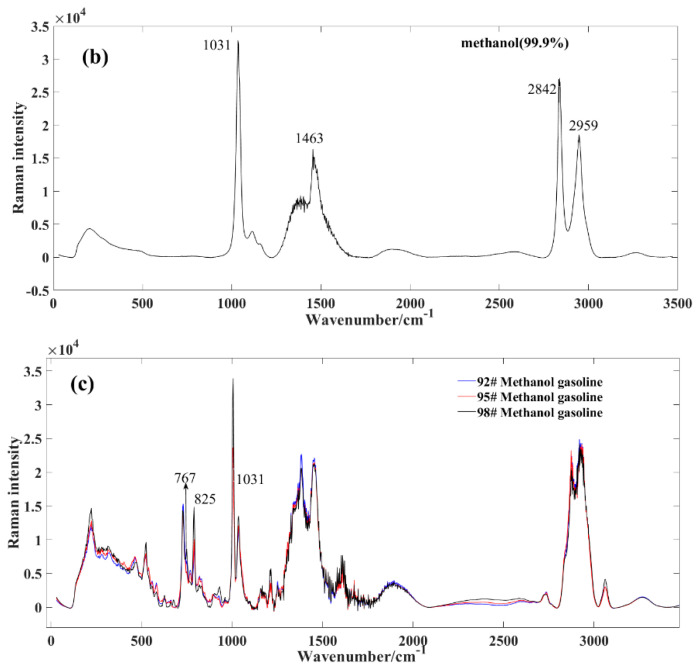
Average spectral profile of gasoline and methanol. (**a**) market gasoline; (**b**) methanol; (**c**) methanol−gasoline blends.

To delineate the distinctive features between methanol, gasoline, and methanol–gasoline blends, we computed average spectra within the range of 0~3500 cm^−1^. Figure 2a shows the average spectrum for three different types of gasoline. The observed variations in gasoline types are primarily attributed to their octane ratings, where higher grades exhibit elevated octane numbers, resulting in intensified peaks within their respective Raman spectra. The composition of gasoline is intricate, characterized by a diverse array of components, each corresponding to distinct Raman spectral characteristic peaks. Even for components sharing the same structure and displaying uniform Raman shifts, variations in peak intensity arise as a result of differing proportions.

By counting, gasoline characterized over 30 distinctive Raman peaks, as depicted in Figure 2a, which highlights the positions of some prominent standard peaks. Among these, the 219 cm^−1^ peak is associated with C-H twisting, the 525 cm^−1^ peak signifies C−C skeleton deformation, the 731 cm^−1^ peak relates to symmetric expansion of the heterogeneous C-C skeleton, and the 793 cm^−1^ peak corresponds to the breathing vibration of naphthenes. Notably, the pronounced Raman spectral peak observed at 1004 cm^−1^ likely associates with the aromatic carbon–carbon double bonds (C=C). Gasoline contains several aromatic hydrocarbon compounds, which typically include aromatic rings containing carbon–carbon double bonds. In Raman spectra, the aromatic C=C bonds often exhibit distinctive Raman peaks, typically occurring around 1000 cm^−1^. Therefore, the significant Raman spectral peak observed at 1004 cm^−1^ may reflect the vibrational mode of aromatic carbon–carbon double bonds present in gasoline. In addition, the peak at 1031 cm^−1^ is likely associated with carbon–hydrogen bonds (C−H), owing to gasoline’s composition primarily comprising hydrocarbons, which are predominantly carbon–hydrogen compounds.

Furthermore, the 1154 cm^−1^ peak represents the antisymmetric vibration of C−C, the 1211 cm^−1^ peak corresponds to the stretching of the phenyl benzene and metaxylene skeletons, the 1383 cm^−1^ peak pertains to diphenyl, the 1455 cm^−1^ peak represents H-C−H, and the 1612 cm^−1^ peak corresponds to toluene and olefin C=C. Additionally, the Raman peak occurring between 2978 cm^−1^ and 2912 cm^−1^ is widely recognized as a characteristic peak of the methylene hydrocarbon group. For a more in-depth exploration of Raman characteristic peaks of gasoline, detailed information can be found in the referenced literature [7,20,23,37,38].

Figure 2b exhibits the Raman spectrum of methanol. In contrast to the complex composition of gasoline, methanol demonstrates relative purity, resulting in a less intricate set of characteristic peaks. The key peak positions for standard samples include: the 1031 cm^−1^ peak, corresponding to the symmetric expansion of the C−H group such as stretching or whirling vibrations; the 1463 cm^−1^ peak, correlating with the CH_2_ torsion + δ (COH); the 2842 cm^−1^ peak, reflecting the symmetric expansion of CH_3_ group; and the 2959 cm^−1^ peak, pertaining to the asymmetric expansion of CH_3_ group [23].

Figure 2c displays the Raman spectrum of methanol–gasoline blends, exhibiting distinct differences in Raman peaks compared to pure gasoline. After mixing methanol with gasoline, we observed a shift in the positions of some spectroscopic peaks, particularly the Raman spectroscopic peaks at 767 and 825 cm^−1^. These peaks shifted with about 32 nm, and this is indicative of the presence of methanol in the blend and provides valuable information about its concentration and interaction with gasoline. At these positions, the intensity of the corresponding peak gradually increases with a rising methanol concentration. From a whole perspective, the peak intensity of methanol–gasoline blends is significantly lower than that of pure gasoline. This is primarily due to the decreasing content of specific gasoline components as the concentration of methanol increases.

### 3.2. Qualitative Analysis of Gasoline

In this section, the principal aim of utilizing the supervised classification method PLS-DA is to evaluate its efficacy in discriminating between methanol–gasoline blend samples and pure gasoline samples. Prior to establishing the PLS-DA model, 96 samples of each type of methanol–gasoline blend model were systematically divided into calibration and prediction sets in a ratio of 2:1. This division resulted in 64 samples for the calibration set and 32 samples for the prediction set. Due to the significant disparity in the number of methanol–gasoline samples compared to pure gasoline samples in the training set, resulting in an imbalance in inter-class sample volumes, the use of PLS-DA classifiers can lead to biased predictions. To address this issue, we employed a simple sample augmentation method, which involved duplicating the spectral data of pure gasoline samples by a factor, thereby equalizing the sample volumes within the two categories.

The classification process unfolds in two key steps: (1) The PLS-DA models were constructed, respectively, utilizing the calibration set for each methanol–gasoline blend. To determine the optimal number of LVs that optimizes the model, a 5-fold cross-validation process was employed. (2) Subsequently, the prediction set was input into the established PLS-DA classification model for prediction, thereby validating the model’s feasibility and assessing its performance on unseen data. This two-step approach ensures a robust evaluation of the ability of the PLS-DA model to effectively classify methanol–gasoline blend samples against pure gasoline samples.

**Figure 3:**
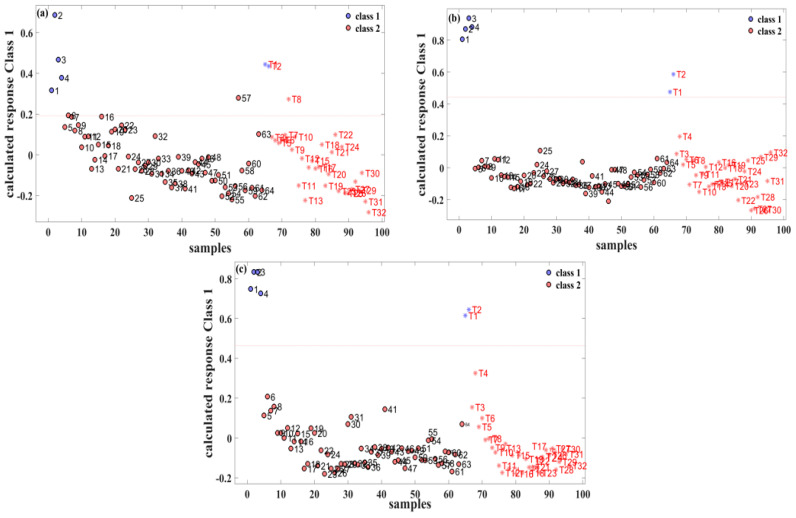
PLS-DA classification results for three different types of methanol–gasoline blend. (**a**) 92# methanol–gasoline blend; (**b**) 95# methanol–gasoline blend; (**c**) 98# methanol–gasoline blend.

The relationship between the sample labels and the calculated responses is vividly depicted in Figure 3. In this representation, “class 1” signifies pure gasoline, while “class 2” represents a methanol–gasoline blend. Specifically, for the 92# methanol–gasoline blend, the optimal number of LVs was determined to be three, resulting in a calibration set prediction accuracy of 98.4% (63/64). There was one misclassification, where the No. 57 methanol–gasoline sample (containing 28% methanol) was classified erroneously as pure gasoline. It is inferred that the acquired spectrum of this sample was caused by manual errors, which led to the error label of prediction. The prediction set achieved a precision of 96.8% (32/33) with one misclassification, wherein the methanol–gasoline sample T8 (with 8% methanol) was mistakenly classified as pure gasoline. Generally, a lower methanol content in methanol–gasoline blends may contribute to classification errors. However, in the cases of No. 57 and T8 samples, where the methanol content is not extremely low, classification errors could potentially be influenced by human factors affecting the Raman spectral results. This nuanced understanding of classification accuracy and potential influencing factors adds depth to the assessment of the performance of the PLS-DA model in distinguishing between pure gasoline and methanol–gasoline blend samples.

For the 95# and 98# methanol–gasoline blends, both the calibration and prediction sets achieved 100% accuracy. The optimal number of LVs for these blends was found to be five and four, respectively, as illustrated in Figure 3b,c. This high accuracy in both calibration and prediction sets underscores the effectiveness of the PLS-DA models for these types of methanol–gasoline blends. The models, with their respective LVs, demonstrate remarkable ability to accurately distinguish between methanol–gasoline blends and pure gasoline samples. This robust performance further emphasizes the reliability and discriminatory power of the PLS-DA approach in the context of different methanol–gasoline blends.

### 3.3. Quantitative Analysis of Methanol in Methanol Gasoline Blends

#### 3.3.1. Variable Selection to Optimize the Model

In order to quantitatively analyze the methanol content in methanol–gasoline blends, the PLS model was initially constructed without selecting useful variables. As shown in Table 1, the correlation coefficients (Rcv and Rp) for both the calibration and prediction sets of the PLS model exceed 0.95. Although the PLS model demonstrates good accuracy, meeting the requirements of daily industrial production, it inherently contains redundant or useless variable information. This surplus information may lead to a decline in model accuracy and robustness. To address this issue, the UVE method was employed to eliminate redundant variable information and enhance the model performance.

The essence of the UVE algorithm is to select the characteristic variables within the spectrum itself by leveraging the statistical insights derived from irrelevant noise variables. A key aspect involves the incorporation or inclusion of random variables, which inherently represent noise. The length of the added noise can be fine-tuned through manual parameter adjustment, with an initial setting typically at half the length of the spectra. Following the addition of noise, the UVE makes a stability judgment on the statistical distribution of the regression coefficient of the target matrix based on the independent variable matrix, which was composed of spectral variables and random noise. The statistical distribution of the regression coefficient is expressed by the ratio of the mean value to the standard deviation. The characteristic variables are finally identified by determining the upper and lower limits and selecting variables falling within the corresponding range.

Due to the random addition of noises in the UVE-PLS procedure, the process was executed 10 times consecutively, and the model yielding the best prediction performance was retained. Among these executions, the number of the remaining variables exhibited some variability, consistently totaling less than 100. Notably, the UVE-PLS effectively discarded the most unstable wavelengths, but the predictive capacity was still close to that of the full-spectra-based PLS model. The selected wavelengths mainly located around the pulse peaks, where there were some organic functional groups [19,23]. Table 1 outlines the number of variables in the multivariate selection model. Through the application of the UVE method for variable selection, the model’s variable count was significantly reduced to less than 5% of that in the PLS model, thereby enhancing operational efficiency. Moreover, the prediction performance of the UVE-PLS model has also shown improvement for all types of methanol–gasoline blends, making it a more efficient and effective tool compared to the traditional PLS model.

#### 3.3.2. Unsupervised Consensus Model

**Figure 4:**
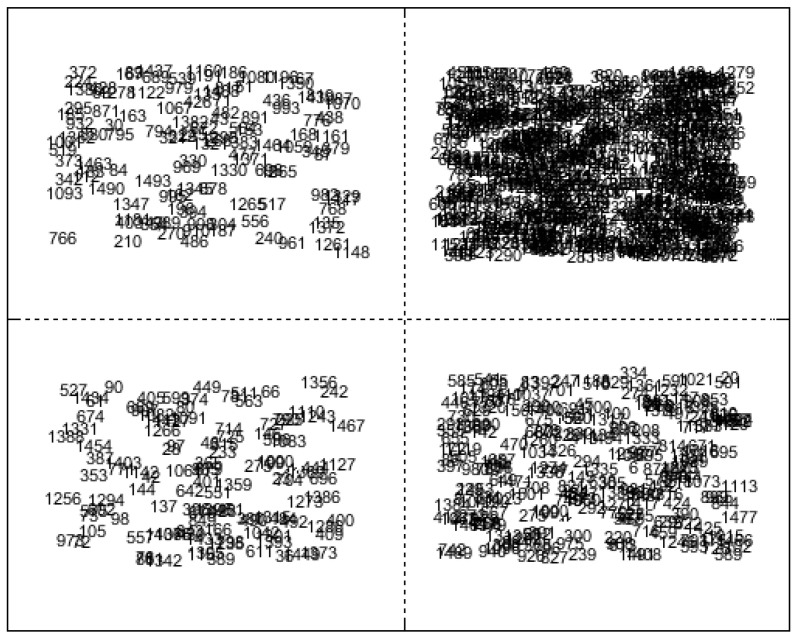
Clustered variables in 2 × 2 array by Kohonen SOM for 92# methanol gasoline blend.

In the development of the unsupervised variable consensus model, the initial step involves applying the SOM algorithm to cluster wavelengths and construct member models. Here, we utilize the SOM algorithm to cluster different types of methanol–gasoline into four clusters based on variable similarity. Wavelengths with similar features were mapped into the same cluster, while those with distinct features were classified into other clusters by the unsupervised SOM network [32]. Consequently, all wavelengths were clustered into *q*^2^ subsets based on their similarities. In the case where ***q*** = 2, the four generated clusters are shown in Figure 4. Labels were numbered (from 1 to 1509) to represent the order of wavelength within the spectral range of 0–3500 cm^−1^. Each clustering unit is considered as *C*_q×q_(*i*,*j*), where *i* indicates the row and *j* the column. *C*_2×2_(1,2) had the most variables, while *C*_2×2_(2,1) had the fewest variables. The selected variables in each cluster unit can be visualized in the original Raman spectrum to observe the distribution of clustered wavelengths. The specific SOM calculation parameters are set as follows: the epoch is two, the neural network comprises two layers, the initial learning rate is one, and the final learning rate is 0.01. Following this clustering, the multi-dimensional spectral variables obtained from these four clusters are individually used to construct corresponding four PLS models (f1, f2, f3, f4), which serve as the member models for the consensus model. The predictive performance of these member models is presented in Table 2. Parameter *q* can be set to various values, allowing for the generation of additional clustering units tailored to the desired regression precision. For illustrative purposes, here we have chosen *q* = 2 as an example.

It is noteworthy that the clusters of multi-dimensional spectral variables generated by the SOM algorithm exhibit significant disparities in the number of variables within clusters, leading to variations in modeling performances. Among these four cluster units, one cluster unit contains more than 200 spectral variables, while another contains nearly 1000 spectral variables, specifically *C*_2×2_(1,2) for 92# methanol–gasoline blends, *C*_2×2_(2,2) for 95# methanol–gasoline blends, and *C*_2×2_(1,1) for 98# methanol–gasoline blends. The other two cluster units contain more than 100 spectral variables each. Their clustering characteristics appear similar, indicating either identical or closely similar spectral profiles.

**Figure 5:**
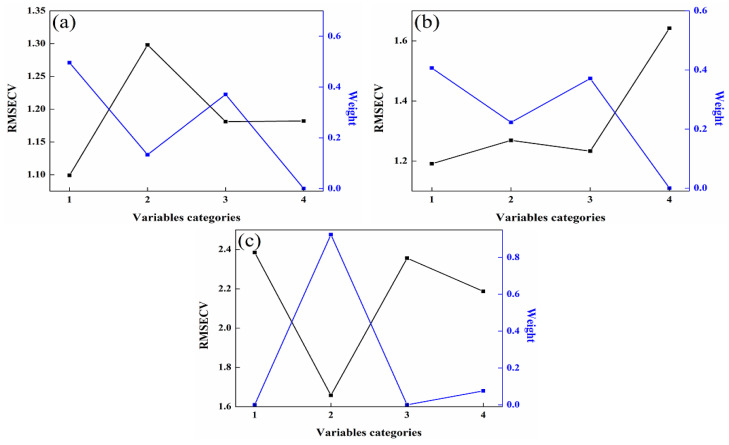
Relationship between weights and RMSECV in the consensus models. (**a**) 92# methanol–gasoline blends; (**b**) 95# methanol–gasoline blends; (**c**) 98# methanol–gasoline blends.

Once the PLS member models are constructed, the unsupervised consensus model is developed by applying the consensus methods outlined in Section 2.4. The weight assigned to each member model can be determined using the Lagrange multiplier method, and this weight value is closely linked to the valuable information contained within each member model. The greater the utility of the variable information, the higher the weight assigned to the member models. As illustrated in Figure 5, the RMSECV value is the smallest for the member model with the highest weight. Consequently, the higher the weight value, the more influential the member model becomes within the unsupervised consensus model. Finally, the unsupervised consensus model is constructed by multiplying the weightings and member models, and its prediction performance is evaluated according to their multiplied values and the measured values. This approach ensures that the most informative member models contribute more significantly to the overall performance of the unsupervised consensus model.

**Figure 6:**
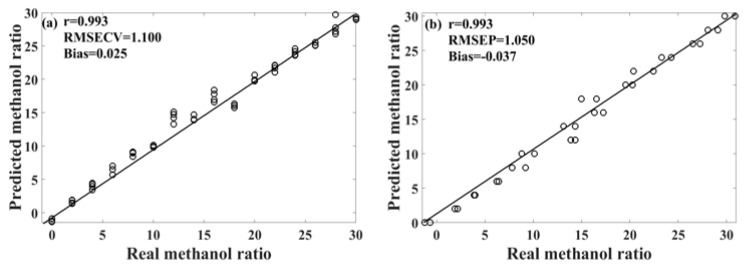
Scatter plot of the predicted results of the unsupervised consensus model for 95# methanol–gasoline. (**a**) Calibration set; (**b**) prediction set.

Table 3 presents the performance of the unsupervised consensus model, showing varying prediction performances for different models of methanol–gasoline blends. These three consensus models demonstrate better prediction performances for methanol–gasoline blends than the original PLS or UVE-PLS models, with both Rcv and Rp exceeding 0.993 for 92# and 95# methanol–gasoline blends. Figure 6 shows the scatter plot of the predicted versus the measured methanol content for 95# methanol–gasoline blends. However, the performance for 98# methanol–gasoline blends is slightly inferior to that of the other two types of blends, although the Rcv and Rp of consensus model are high, reaching 0.984, outperforming the PLS or UVE-PLS models. Further observation finds that the difference between RMSECV and RMSEP in the 98# blends is larger than in others, indicating a risk of overfitting associated with the selection of modeling parameters or the imbalance of the spectral variation between the calibration set and the prediction set. In contrast, the RMSECV/RMSEP ratios for 92# and 95# methanol–gasoline are close to one, respectively, indicating satisfactory modeling results. Therefore, there is still room for improvement in the unsupervised consensus model established for 98# methanol–gasoline blends. Further refinement may enhance its predictive accuracy and mitigate the risk of overfitting.

### 3.4. Discussions

In this work, Raman spectroscopy coupled with the PLS-DA model was employed to qualitatively analyze various types of methanol–gasoline blends and gasoline. According to the model evaluation criteria, the PLS-DA models developed for different methanol–gasoline blends have exhibited satisfactory performances. In particular, the accuracy achieved for 95# and 98# ethanol–gasoline blends reached 100%. This high level of accuracy underscores the practical and promotional potential of the PLS-DA model in qualitative analysis of methanol–gasoline blends.

For the quantitative analysis of methanol–gasoline blends, we systematically developed the unsupervised consensus model, the full-spectral-based PLS model, and the supervised multivariate selection model (UVE-PLS), aiming to determine the best prediction model through comparative analysis of their performances. The results, as presented in Table 1, demonstrate the improvements in the prediction performance of the UVE-PLS model compared to the PLS model. Through the comprehensive comparison and analysis, it was observed that the prediction performance of the UVE-PLS model can be effectively enhanced by employing the multivariate selection method to extract valuable information from the entire spectrum. Moreover, utilizing UVE to select useful variable information significantly reduces the number of variables required for model development, thereby enhancing model robustness and operational efficiency. Thus, it can be concluded that the multivariate selection method effectively simplifies the computational complexity of the model. Compared to the supervised UVE-PLS modeling approach, the unsupervised consensus model integrates the member model developed using the SOM algorithm based on variable similarity. Each member model contributes to the unsupervised consensus model according to its weight, reducing the reliance on any single member model. Despite the poorer prediction performance of individual member models compared to the UVE-PLS model, the unsupervised consensus model outperforms the UVE-PLS model, leveraging the useful variable information from member models while mitigating the interference of irrelevant variable information. As evident from Table 2 and Table 3, the prediction performance of the unsupervised consensus model is more robust and stable compared to its member models, effectively addressing overfitting issues. Thus, it can be inferred that the consensus modeling strategy enhances the prediction performance of the unsupervised clustering SOM-assisted model.

Methanol–gasoline blends, recognized as a globally promoted biofuel, require comprehensive qualitative and quantitative analysis for quality control and monitoring. In this study, we successfully devised PLS-DA and unsupervised consensus models based on Raman technology for the analysis of methanol–gasoline blends. These models provide an efficient detection strategy and serve as valuable references for future detection methodologies. However, it is essential to note that the models developed in this study have certain limitations, such as relatively high RMSECV and RMSEP values, and the potential risk of overfitting in some methanol–gasoline models. Following a comprehensive comparison, it was determined that the unsupervised consensus model, specifically constructed using 95# methanol–gasoline, exhibited the most favorable prediction performance, as evidenced by the scatter plot depicted in Figure 6. In future studies, we can further increase the number of SOM clustering units and then employ consensus modeling to screen effective member models. This approach aims to reduce spectral variables and optimize the model, ultimately enhancing its performance and predictive accuracy.

## 4. Materials and Methods

### 4.1. Sample Preparation

In this study, the methanol–gasoline blends were meticulously prepared through artificial means, beginning with the initial market samples. Commercially available methanol–gasoline blends were served as the reference base mixture. This base was then combined with three different types of gasoline (92#, 95#, and 98# sourced from six local gas stations in Hefei City, China) and anhydrous methanol (supplied Aladdin Reagent Inc., Product No. M116122) within a laboratory setting. The blending process involved varying the volume percentages of methanol, ranging from 2% to 30%, with 2% intervals (comprising a total of 16 gradients within the 0% to 30% methanol range). This systematic approach resulted in the formulation of 288 unique blends (=16 gradients × 6 stations × 3 gasolines), derived from the combination of three gasoline types, six gas stations, and 16 methanol gradients. To ensure homogeneity and prevent liquid desalination, the mixtures underwent thorough oscillation. Subsequently, the configured methanol–gasoline blends were transferred into centrifuge tubes in preparation for the subsequent spectral scanning process.

### 4.2. Collection of Raman Spectra

Spectral data for three distinct methanol–gasoline blends were obtained using a RK785-III Raman spectrometer (Shanghai Ruhai Optoelectronics Technology Co., Ltd., Shanghai, China). Spectra were collected in a spectral range spanning from 0 cm^−1^ to 3500 cm^−1^, with a resolution set at 3 cm^−1^. Each blend, contained within a centrifuge tube, was subjected to three repetitive scans with minor positional adjustments. The resulting spectra from these scans were then averaged to yield the final spectral representation of each blend sample. This meticulous process ensured a reliable and representative characterization of the Raman spectra for the methanol–gasoline blends under investigation.

**Figure 7:**
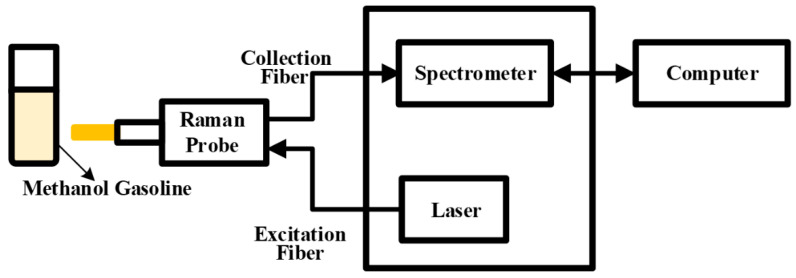
Schematic diagram of the Raman acquisition system.

As depicted in Figure 7, this experiment utilized a Raman spectroscopy acquisition system. The system comprises two primary components: the Raman probe and the Raman acquisition host, which encompasses a dispersion fiber spectrometer and a 785 nm semiconductor laser. The configured sample, a mixture of methanol and gasoline, was placed within a 1.5 mm quartz cuvette. The semiconductor laser transmitted the laser beam to the Raman probe via the optical fiber. Upon irradiation of the methanol–gasoline mixture by the laser, a Raman signal was generated and captured by the Raman probe. The acquired signal then traversed across the transmission fiber to reach the dispersive fiber spectrometer. Within the spectrometer, the Raman scattered light was separated and sampled. Subsequent to analog-to-digital (A/D) conversion, the spectral information became accessible and was transmitted to the computer through the I/O data port. This integrated system facilitated the efficient acquisition and analysis of Raman spectra from the methanol–gasoline sample.

### 4.3. Evaluation of Models Performance

In qualitative analysis, the PLS-DA model commonly employs “accuracy” as a key indicator to assess its classification performance. In the realm of quantitative analysis, whether utilizing a multivariate selection model or an unsupervised consensus model, various metrics such as root mean square error of cross-validation (RMSECV), root mean square error of prediction (RMSEP), correlation coefficients (Rcv, Rp), Bias, etc., serve as indicators for evaluating the quality of the model. Generally, a good calibration model should have small parameters of RMSE and bias (tends to 0), and a big R (left tends to 1), but a small difference between the RMSECV and the RMSEP, as well as the Rcv and Rp, which indicate the performance of the model in the calibration stage and prediction stage, respectively.

In the consensus model, RMSECV is calculated by actual *y* and consensual *F*(*x*), which is a linear combination of the predictor *y_i_* in the cross-validation stage from the member model *f_i_*(*x_i_*), as well as Rcv. These calculations were executed using MATLAB 2018a (The Math Works, Natick, MA, USA), providing a robust computational environment for the evaluation of model performance in both qualitative and quantitative analyses.

### 4.4. Spectral Pretreatments

During the collection of Raman spectra for methanol–gasoline blends, susceptibility to external factors introduces potential interference. The acquired spectrum may encompass extraneous information unrelated to the tested sample, such as random noise arising from human operation of the detection instrument and fluorescence generated by fluorescent substances within the sample. Consequently, preprocessing of the original spectrum becomes imperative with the primary goal of mitigating the impact of these interference factors. Commonly employed preprocessing methods for Raman spectral data include smoothing, derivation, and normalization, among others. This method was chosen to effectively treat the Raman spectra, enhancing the signal-to-noise ratio and facilitating a more accurate representation of the sample-specific information by reducing the impact of undesired interference factors.

## 5. Conclusions

In this study, we utilized Raman spectroscopy for both qualitative and quantitative analyses of methanol content in methanol–gasoline blends. Our calculations yielded highly precise and reliable results, validating the efficacy of Raman spectroscopy in this context. In our qualitative analysis, PLS-DA was employed to model and analyze various methanol–gasoline blends, achieving notably high accuracy levels. Particularly, both the calibration and prediction sets for 95# and 98# methanol–gasoline blends achieved 100% accuracy.

In the realm of quantitative analysis, we constructed the unsupervised consensus model along with its reference model, the supervised multivariate selection model (UVE-PLS). Compared to the UVE-PLS model, the unsupervised consensus model optimally leveraged valuable information within its member models, resulting in superior final prediction performance. This research underscores the vast potential of Raman technology in fuel–oil composition analysis and emphasizes the need for the robust development of Raman spectroscopy’s application value in this domain. The combination of qualitative and quantitative approaches shows the versatility and efficacy of Raman spectroscopy in understanding and predicting the composition of complex fuel–oil mixtures.

## Figures and Tables

**Table 1:** Quantitative analysis of the methanol content in methanol gasoline blends.

Sample	Modeling Methods	N	LVs	Calibration Set			Prediction Set		
				RMSECV	Rcv	Bias	RMSEP	Rp	Bias
92#	PLS	1509	3	1.100	0.993	0.020	1.366	0.990	−0.274
95#	PLS	1509	3	1.296	0.990	0.004	1.158	0.993	−0.142
98#	PLS	1509	5	2.299	0.969	0.018	2.662	0.959	0.287
92#	UVE-PLS	20	4	1.080	0.993	0.004	1.320	0.991	−0.359
95#	UVE-PLS	28	4	1.092	0.993	−0.010	1.125	0.992	−0.130
98#	UVE-PLS	46	5	2.139	0.973	0.037	2.211	0.971	0.222

Note: N: the number of involving wavelengths; LVs: latent variables in PLS model; PLS: partial least squares regression model; UVE: uninformative variable elimination.

**Table 2:** Prediction performances of PLS models based on clustered variables.

Sample	Modeling Methods	N	LVs	Calibration Set			Prediction Set		
				RMSECV	Rcv	Bias	RMSEP	Rp	Bias
92#	*f*_1_	132	7	1.099	0.993	0.050	1.214	0.992	−0.205
92#	*f*_2_	1010	7	1.298	0.990	0.093	1.457	0.988	0.408
92#	*f*_3_	107	5	1.131	0.993	0.014	1.275	0.991	0.342
92#	*f*_4_	260	8	1.183	0.992	0.145	1.271	0.991	0.311
95#	*f*_1_	242	6	1.191	0.992	−0.035	1.154	0.992	0.086
95#	*f*_2_	110	4	1.269	0.991	−0.017	1.391	0.989	0.008
95#	*f*_3_	162	4	1.233	0.991	−0.021	1.090	0.994	0.001
95#	*f*_4_	995	5	1.642	0.984	0.027	1.317	0.991	−0.042
98#	*f*_1_	1008	8	2.385	0.965	0.040	2.411	0.966	−0.035
98#	*f*_2_	244	10	1.659	0.984	0.116	2.160	0.974	0.301
98#	*f*_3_	145	4	2.357	0.967	0.012	2.404	0.966	0.001
98#	*f*_4_	112	4	2.187	0.972	−0.002	2.331	0.969	0.260

Note: N is the number of variables included in the member model. LVs is the number of latent variables in PLS model. fi is the PLS member model developed with the *i*th clustered spectral variables. The bold one is the best among developed four member models.

**Table 3:** Predicted performance of the unsupervised consensus model.

Sample	Relation	Calibration Set			Prediction Set		
		RMSECV	Rcv	Bias	RMSEP	Rp	Bias
92# methanol gasoline	a	1.052	0.994	−0.043	1.194	0.994	0.283
95# methanol gasoline	b	1.101	0.993	0.025	1.050	0.993	−0.037
98# methanol gasoline	c	1.653	0.984	−0.107	2.144	0.984	−0.298

Note: a = 0.4959 × *f*_1_ + 0.1332 × *f*_2_ + 0.3708 × *f*_3_ + 0.0001 × *f*_4_; b = 0.4066 × *f*_1_ + 0.2225 × *f*_2_ + 0.3709 × *f*_3_ + 0.0000 × *f*_4_; c = 0.0000 × *f*_1_ + 0.9241 × *f*_2_ + 0.0000 × *f*_3_ + 0.0759 × *f*_4_.

## Data Availability

The data presented in this study are available in the article and Supplementary Materials.

## Supplementary Materials

The following supporting information can be downloaded at: https://www.mdpi.com/article/10.3390/molecules29071427/s1. Table S1. The methanol content in the methanol-gasoline blends.
