# Supplementary material for: Unsupervised Clustering-Assisted Method for Consensual Quantitative Analysis of Methanol–Gasoline Blends by Raman Spectroscopy
Source: Molecules. 2024 Mar 22;29(7):1427. doi: 10.3390/molecules29071427 (PMC11013198; doi:10.3390/molecules29071427)
Supplement: Supplementary file 1 [file molecules-29-01427-s001.zip › molecules-2866005-supplementary.pdf]

Table S1. The methanol content in the methanol-gasoline blends listed as follows:

gas station	92#	95#	98#
1	0%, 2%, 4%,..., 30%.	0%, 2%, 4%,..., 30%.	0%, 2%, 4%,..., 30%.
2	0%, 2%, 4%,..., 30%.	0%, 2%, 4%,..., 30%.	0%, 2%, 4%,..., 30%.
3	0%, 2%, 4%,..., 30%.	0%, 2%, 4%,..., 30%.	0%, 2%, 4%,..., 30%.
4	0%, 2%, 4%,..., 30%.	0%, 2%, 4%,..., 30%.	0%, 2%, 4%,..., 30%.
5	0%, 2%, 4%,..., 30%.	0%, 2%, 4%,..., 30%.	0%, 2%, 4%,..., 30%.
6	0%, 2%, 4%,..., 30%.	0%, 2%, 4%,..., 30%.	0%, 2%, 4%,..., 30%.
